# Synthesis and styrene copolymerization of novel trisubstituted ethylenes: 3. Alkoxy ring-substituted isopropyl 2-cyano-3-phenyl-2-propenoates

**DOI:** 10.1080/15685551.2018.1531961

**Published:** 2018-10-15

**Authors:** Gregory B. Kharas, Nita Shinde, Jasmine K. Jody, Emily K. Mosher, Navneet Kaur, Anige’r A.R. Oriol, Destiny M. Perez, Raj Ranganathan, Ashley Renteria, Taryn A. Rydbom, Caroline Yeager, William S. Schjerven

**Affiliations:** Chemistry Department, DePaul University, Chicago

**Keywords:** Trisubstituted ethylenes, styrene copolymers, radical copolymerization

## Abstract

Novel trisubstituted ethylenes, alkoxy ring-substituted isopropyl 2-cyano-3-phenyl-2-propenoates, RPhCH = C(CN)CO_2_CH(CH_3_)_2_ (where R is 2-methoxy, 3-methoxy, 4-methoxy, 2-ethoxy, 3-ethoxy, 4-ethoxy, 4-propoxy, 4-butoxy, 4-hexyloxy) were prepared and copolymerized with styrene. The ethylenes were synthesized by the piperidine catalyzed Knoevenagel condensation of ring-substituted benzaldehydes and isopropyl cyanoacetate, and characterized by CHN analysis, FTIR, ^1^H and ^13^C NMR. All the ethylenes were copolymerized with styrene in solution with radical initiation (ABCN) at 70°C. The compositions of the copolymers were calculated from nitrogen analysis and the structures were analyzed by FTIR, ^1^H and ^13^C NMR. Decomposition of the copolymers in nitrogen occurred in two steps, first in the 250-500ºC range with residue (2.6–3.9% wt.), which then decomposed in the 500-800ºC range.

## 1. Introduction

Ring–functionalized trisubstituted ethylenes (TSE), esters of 2-cyano-3-phenyl-2-propenoic acid, R^1^PhCH = C(CN)CO_2_R^2^ continue to attract attention as compounds with interesting properties and as comonomers for modification of commercial polymers. There are application reports exemplifying methyl 2-cyano-3-phenyl-2-propenoate, MCPP [1,2]. Thus, ring-unsubstituted MCPP was employed in studies of beta amino acid-modified and fluorescently labeled kisspeptin analogues with potent KISS1R activity [1] and in reversible targeting of noncatalytic cysteines with chemically tuned electrophiles [2]. Methoxy ring-substituted MCPP was used in photorefractive polymer composites [3] as well as in synthesis of new triazoles for potential antifungal agents applications [4].

Methoxy ring-substituted ethyl 2-cyano-3-phenyl-2-propenoate, ECPP was prepared by condensation catalyzed by the L-lysine functionalized polyacrylonitrile fiber [5]. Methoxy ring-substituted propyl 2-cyano-3-phenyl-2-propenoate, PCPP was the product of metal-free synthesis via cyanuric chloride-mediated three-component reactions involving a cascade consists of Knoevenagel condensation/cyano hydration/esterification [6].

It was shown that electrophilic tri- and tetrasubstituted ethylenes are particularly useful in delineating the transition from radical chemistry to ionic chemistry [7]. In regards to polymerization reactivity, previous studies showed that TSE containing substituents larger than ﬂuorine have very low reactivity in radical homopolymerization due to polar and steric reasons. Although steric difﬁculties preclude homopolymerization of most TSE monomers, their copolymerization with a monosubstituted alkene makes it possible to overcome these steric problems [8]. Copolymerization of electrophylic TSE compounds having double bonds substituted with halo, cyano, and carbonyl groups and electron-rich monosubstituted ethylenes such as styrene, *N*-vinylcarbazole, and vinyl acetate [9–11] show a tendency toward the formation of alternating copolymers – thus suggesting a way of functionalization of commercial polymers via introduction of isolated TSE monomer units.

With the objective to design unique structures, that could serve as a spring board for further development of novel compounds and materials with new properties and applications we have reported synthesis and styrene copolymerization of alkoxy ring-substituted methyl [12–14], ethyl [15,16], propyl [17], and butyl [18] esters of 2-cyano-3-phenyl-2-propenoates. Our purposes in exploration of novel isopropyl 2-cyano-3-phenyl-2-propenoates (ICPP) were twofold: (1) to utilize aldol condensation for synthesis of alkenes with a variety of potentially reactive functional groups; (2) to employ conventional radical copolymerization of novel functional comonomers with a commercial monomer styrene.

Thus, in continuation of our investigation of novel TSE compounds [19] we have prepared alkoxy ring-substituted isopropyl 2-cyano-3-phenyl-2-propenoates, ICPP, RPhCH = C(CN)CO_2_CH(CH_3_)_2_, where R is 2-methoxy, 3-methoxy, 4-methoxy, 2-ethoxy, 3-ethoxy, 4-ethoxy, 4-propoxy, 4-butoxy, 4-hexyloxy, and explored the feasibility of their copolymerization with styrene. To the best of our knowledge, except for synthesis of 2-methoxy, 3-methoxy, and 4-methoxy ring-substituted isopropyl 2-cyano-3-phenyl-2-propenoates [20] there have been no reports on either synthesis of these compounds, nor their copolymerization with styrene.

## 2. Experimental

### 2.1. General procedures

Infrared spectra of the ICPP compounds and polymers (NaCl plates) were determined with an ABB FTLA 2000 FTIR spectrometer. The melting points of the ICPP compounds and the glass transition temperatures (*T*_g_), of the copolymers were measured with TA (Thermal Analysis, Inc.) Model Q10 differential scanning calorimeter (DSC). The thermal scans were performed in a 25 to 150ºC range on second heat at heating rate of 10ºC/min. *T*_g_ was taken as a midpoint of a straight line between the inflection of the peak’s onset and endpoint. The thermal stability of the copolymers was measured by thermogravimetric analyzer (TGA) TA Model Q50 from ambient temperature to 800ºC at 20ºC/min in the flow of nitrogen (20 mL/min). The molecular weights of the polymers was determined relative to polystyrene standards in THF solutions with sample concentrations 0.8% (w/v) by gel permeation chromatography (GPC) using a Altech 426 HPLC pump at an elution rate of 1.0 mL/min; Phenogel 5μ Linear column at 25ºC and Viscotek 302 RI detector. ^1^H and ^13^C NMR spectra were obtained on 10–25% (w/v) ICPP or polymer solutions in CDCl_3_ at ambient temperature using Avance 300 MHz spectrometer. Elemental analyses of ICPP compounds and the copolymers were performed by Midwest Microlab, LLC (IN).

### 2.2. Synthesis and characterization of isopropyl 2-cyano-3-phenyl-2-propenoates

2-Methoxy (≥97.5%), 3-methoxy (98%), 4-methoxy (98%), 2-ethoxy (98%), 3-ethoxy(95%), 4-ethoxy (96%), 4-propoxy (≥97%), 4-butoxy (95%), hexyloxy (96%) substituted benzaldehydes, isopropyl cyanoacetate (≥98.0%), piperidine (99%) supplied from Sigma-Aldrich Co., were used as received. The preparation procedure was essentially the same for all the monomers. In a typical synthesis, equimolar amounts of isopropyl cyanoacetate and an appropriate benzaldehyde were mixed in equimolar ratio in a 20 mL vial. A few drops of piperidine were added with stirring. The product of the reaction was isolated by ﬁltration and puriﬁed by crystallization from 2-propanol. The condensation reaction proceeded smoothly, yielding products, which were puriﬁed by conventional techniques. Melting points of the compounds in crystalline state were measured by DSC. The compounds were characterized by FTIR, ^1^H and ^13^C NMR spectroscopies. No stereochemical analysis of the novel alkoxy ring-substituted ICPP was performed since no stereoisomers (*E* or/and *Z*) of known configuration were available.

#### 2.2.1. Isopropyl 2-cyano-3-(2-methoxyphenyl)-2-propenoate

Yield: 78.0%; mp 72.8°C; ^1^H NMR: *δ* 8.7 (s, 1H, CH = ), 8.3–6.9 (m, 4H, Ph), 5.3 (m, 1H, CH), 3.9 (s, 3H, CH_3_O), 1.3 (d, 6H, CH_3_); ^13^C NMR: *δ* 164 (C = O), 152 (HC = ), 135, 131, 130, 122 (Ph), 116 (CN), 111 (C = ), 68 (CH), 56 (OCH_3_), 22 (CH_3_); FTIR: (cm^−1^) 3064–2821 (m, C-H), 2224 (m, CN), 1705 (s, C = O), 1597 (s, C = C), 1278 (s, C-O-CH_3_), 769, 710 (s, C-H out of plane). Anal. calcd. for C_14_H_15_NO_3_: C, 68.56; H, 6.16; N, 5.71; Found: C, 68.71; H, 6.48; N, 5.84.

#### 2.2.2. Isopropyl 2-cyano-3-(3-methoxyphenyl)-2-propenoate

Yield 82%; ^1^H NMR *δ* 8.6 (s, 1H, CH = ), 8.1–7.2 (m, 4H, Ph), 5.1 (m, 1H, CH), 3.9 (s, 3H, CH_3_O), 1.4 (d, 6H, (CH_3_)_2_); ^13^C NMR *δ* 167 (C = O), 152 (HC = ), 131, 130, 126 (Ph), 116 (CN), 104 (C = ), 68 (CH), 56 (OCH_3_), 22 (CH_3_); IR (cm^−1^): 3082–2828 (m, C-H), 2223 (m, CN), 1734 (s, C = O), 1223 (s, C-O-CH_3_), 882, 788 (s, C-H out of plane). Anal. Calcd. for C_14_H_15_NO_3_: C, 68.56; H, 6.16; N, 5.71; Found: C, 67.44; H, 6.27; N, 5.66.

#### 2.2.3. Isopropyl 2-cyano-3-(4-methoxyphenyl)-2-propenoate

Yield 89%; mp 71.8°C; ^1^H NMR *δ* 8.2 (s, 1H, CH = ), 7.9–7.0 (m, 4H, Ph), 5.1 (m, 1H, CH), 3.9 (s, 3H, OCH_3_), 1.3 (d, 6H, CH_3_); ^13^C NMR *δ* 166 (C = O), 154 (HC = ), 138, 136, 132, 131, 130, 129 (Ph), 116 (CN), 102 (C = ), 68 (CH), 56 (OCH_3_), 22 (CH_3_); FTIR (cm^−1^): 3087–2839 (m, C-H), 2220 (m, CN), 1707 (s, C = O), 1228 (s, C-O-CH_3_), 897 (s, C-H out of plane). Anal. Calcd. for C_14_H_15_NO_3_: C, 68.56; H, 6.16; N, 5.71; Found: C, 68.27; H, 6.25; N, 5.67.

#### 2.2.4. Isopropyl 2-cyano-3-(2-ethoxyphenyl)-2-propenoate

Yield 81%; mp 55.3°C; ^1^H NMR *δ* 8.8 (s, 1H, CH = ), 8.3–6.8 (m, 4H, Ph), 5.2 (m, 1H, CH), 4.2 (q, 2H, CH_2_), 1.5 (t, 3H, CH_3_), 1.4 (d, 6H, (CH_3_)_2_); ^13^C NMR *δ* 166 (C = O), 152 (HC = ), 134, 131, 130, 125, 112 (Ph), 116 (CN), 111 (C = ), 68 (CH), 64 (CH_2_), 22 (CH_3_)_2_, 15 (CH_3_); FTIR (cm^−1^): 3074–2829 (m, C-H), 2222 (m, CN), 1722 (s, C = O), 1597 (s, C = C), 1246 (s, C-O-CH_3_), 896 (s, C-H out of plane). Anal. Calcd. for C_15_H_17_NO_3_: C, 69.48; H, 6.61; N, 5.40; Found: C, 69.53; H, 6.66; N, 5.48.

#### 2.2.5. Isopropyl 2-cyano-3-(3-ethoxyphenyl)-2-propenoate

Yield 95%; mp 82.9°C; ^1^H NMR *δ* 8.2 (s, 1H, CH = ), 7.7–7.1 (m, 4H, Ph), 5.3 (m, 1H, CH), 4.2 (q, 2H, CH_2_), 1.4 (d, 6H, CH_3_), 1.2 (t, 3H, CH_3_); ^13^C NMR *δ* 166 (C = O), 152 (HC = ), 135, 131, 129, 125 (Ph), 116 (CN), 111 (C = ), 68 (CH), 64 (CH_2_), 22 (CH_3_)_2_, 15 (CH_3_); FTIR (cm^−1^): 3078–2834 (m, C-H), 2224 (m, CN), 1728 (s, C = O), 1556 (C = C), 1265 (s, C-O-CH_3_), 842 (s, C-H out of plane). Anal. Calcd. for C_15_H_17_NO_3_: C, 69.48; H, 6.61; N, 5.40; Found: C, 67.59; H, 6.78; N, 5.54.

#### 2.2.6. Isopropyl 2-cyano-3-(4-ethoxyphenyl)-2-propenoate

Yield 86%; mp 87.1°C; ^1^H NMR *δ* 8.2 (s, 1H, CH = ), 8.1–7.2 (m, 4H, Ph), 5.2 (m, 1H, CH), 4.2 (q, 2H, CH_2_), 1.5 (t, 3H, CH_3_), 1.4 (d, 6H, CH_3_); ^13^C NMR *δ* 164 (C = O), 154 (HC = ), 138, 132, 122, 112 (Ph), 116 (CN), 107 (C = ), 68 (CH), 64 (CH_2_), 22 (CH_3_)_2_, 14 (CH_3_); FTIR (cm^−1^): 3035–2805 (m, C-H), 2218 (m, CN), 1713 (s, C = O), 1590 (C = C), 1254 (s, C-O-CH_3_), 846 (s, C-H out of plane). Anal. Calcd. for C_15_H_17_NO_3_: C, 69.48; H, 6.61; N, 5.40; Found: C, 69.37; H, 6.77; N, 5.35.

#### 2.2.7. Isopropyl 2-cyano-3-(4-propoxyphenyl)-2-propenoate

Yield 79%; mp 70.4°C; ^1^H NMR *δ* 8.2 (s, 1H, CH = ), 8.1–7.2 (m, 4H, Ph), 5.1 (m, 1H, CH), 3.9 (t, 2H, CH_2_O), 1.7 (m, 2H, CH_2_CH_2_O), 1.3 (d, 6H, CHCH_3_), 1.2 (d, 3H, CH_3_CH_2_CH_2_Ph); ^13^C NMR *δ* 166 (C = O), 152 (HC = ), 135, 131, 130, 122, 112 (Ph), 116 (CN), 111 (C = ), 70 (CH_3_CH_2_CH_2_O), 68 (OCH), 23 (CH_3_CH_2_CH_2_O), 22 (OCHCH_3_), 10 (OCH_2_CH_2_CH_3_); FTIR (cm^−1^): 3035–2805 (m, C-H), 2216 (m, CN), 1713 (s, C = O), 1521 (C = C), 1254 (s, C-O-CH_3_), 846 (s, C-H out of plane). Anal. Calcd. for C_16_H_19_NO_3_: C, 70.31; H, 7.01; N, 5.12; Found: C, 70.38; H, 7.12; N, 5.16.

#### 2.2.8. Isopropyl 2-cyano-3-(4-butoxyphenyl)-2-propenoate

Yield 58%; mp 72.5°C; ^1^H NMR *δ* 8.2 (s, 1H, CH = ), 8.0, 7.0 (m, 4H, Ph), 5.2 (t, 1H, OCH), 4.1 (t, 2H, C_3_H_7_CH_2_O), 1.8 (m, 2H, C_2_H_5_CH_2_CH_2_O), 1.5 (m, 2H, CH_3_CH_2_CH_2_CH_2_O), 1.4 (d, 6H, CH_3_), 1.0 (t, 3H, CH_3_CH_2_CH_2_CH_2_O); ^13^C NMR *δ* 166 (C = O), 152 (HC = ), 138, 131, 130, 122, 112 (Ph), 116 (CN), 111 (C = ), 69 (C_3_H_7_CH_2_O), 68 (CH), 31 (C_2_H_5_CH_2_CH_2_O), 22 (CH_3_)_2_, 20 (CH_3_CH_2_CH_2_CH_2_O), 14 (CH_3_CH_2_CH_2_CH_2_O); FTIR (cm^−1^): 3034–2808 (m, C-H), 2216 (m, CN), 1711 (s, C = O), 1585 (C = C), 1256 (s, C-O-CH_3_), 837 (s, C-H out of plane). Anal. Calcd. for C_17_H_21_NO_3_: C, 71.06; H, 7.37; N, 4.87; Found: C, 70.83; H, 7.28; N, 4.91.

#### 2.2.9. Isopropyl 2-cyano-3-(4-hexyloxyphenyl)-2-propenoate

Yield 64.0%; mp 56°C; ^1^H NMR *δ* 8.8 (s, 1H, CH = ), 7.8, 6.9 (m, 4H, Ph), 4.8 (m, 1H, CH), 4.3 (t, 2H, CH_2_O), 1.8 (m, 2H, CH_2_CH_2_O), 1.5 (d, 6H, CH_3_), 1.4 (m, 2H, CH_2_CH_2_CH_2_O), 1.3 (m, 2H, CH_2_CH_2_CH_2_CH_2_O), 1.2 (m, 2H, CH_2_CH_2_CH_2_CH_2_CH_2_O), 0.9 (t, 3H, CH_3_CH_2_); ^13^C NMR *δ* 166 (C = O), 152 (HC = ), 135, 131, 130, 122 (Ph), 116 (CN), 111 (C = ), 69 (CH_2_O), 68 (CH), 31(CH_2_CH_2_CH_2_CH_2_O), 29 (CH_2_CH_2_O), 26 (CH_2_CH_2_CH_2_O), 22 (CH_3_CH_2_), 21 (CHCH_3_), 14 (CH_3_CH_2_); FTIR (cm^−1^): 3056–2808 (m, C-H), 2222 (m, CN), 1720 (s, C = O), 1585 (C = C), 1245 (s, C-O-CH_3_), 924 (s, C-H out of plane). Anal. Calcd. for C_19_H_25_NO_3_: C, 72.35; H, 7.99; N, 4.44; Found: C, 72.32; H, 7.90; N, 4.39.

### 2.3. Synthesis of styrene – ICPP copolymers

Styrene (≥99%), 1,1ʹ-azobiscyclohexanecarbonitrile (98%), (ABCN), and toluene (98%) supplied from Sigma-Aldrich Co., were used as received. Copolymers of the ST and the ICPP compounds, P(ST-co-ICPP) were prepared in 25-mL glass screw cap vials at ST/ICPP = 3 (mol) the monomer feed using 0.12 mol/L of ABCN at an overall monomer concentration 2.44 mol/L in 10 mL of toluene. The copolymerization was conducted at 70ºC. After 5 hours the mixture was cooled to room temperature, and precipitated dropwise in methanol. The composition of the copolymers was determined based on the nitrogen content. The compounds were characterized by FTIR, ^1^H and ^13^C NMR spectroscopies. Thermal behavior was studied by DSC and TGA.

#### 2.3.1. Styrene – isopropyl 2-cyano-3-(2-methoxyphenyl)-2-propenoate copolymer

Yield: 16.2%; ^1^H NMR: *δ* 7.6–6.0 (Ph), 5.2–4.9 (CHCH_3_), 4.3–3.6 (CH_3_O), 3.8–2.2 (CH), 3.7–1.6 (CH_2_), 1.1–0.4 (CH_3_); ^13^C NMR: *δ* 165–163 (C = O), 143–121 (Ph), 118–110 (CN), 71–68 (CH), 60–53 (OCH_3_), 45–40 (CH_2_) 39–32 (CH), 22–18 (CH_3_); FTIR: (cm^−1^) 3064–2821 (m, C-H), 2231 (m, CN), 1734 (s, C = O), 1246 (s, C-O-CH_3_), 789 (s, C-H out of plane). Anal. for N, 2.70.

#### 2.3.2. Styrene – isopropyl 2-cyano-3-(3-methoxyphenyl)-2-propenoate copolymer

Yield 12.3%; ^1^H NMR *δ* 7.6–6.0 (Ph), 5.2–4.9 (CHCH_3_), 4.3–3.6 (CH_3_O), 3.8–2.2 (CH), 3.7–1.6 (CH_2_), 1.1–0.4 (CH_3_); ^13^C NMR: *δ* 165–163 (C = O), 143–121 (Ph), 118–110 (CN), 71–68 (CH), 60–53 (OCH_3_), 45–40 (CH_2_) 39–32 (CH), 22–18 (CH_3_); FTIR (cm^−1^): 3087–2839 (m, C-H), 2240 (m, CN), 1732 (s, C = O), 1228 (s, C-O-CH_3_), 928 (s, C-H out of plane). Anal. for N, 2.00.

#### 2.3.3. Styrene – isopropyl 2-cyano-3-(4-methoxyphenyl)-2-propenoate copolymer

Yield 13.2%; ^1^H NMR *δ* 7.6–6.0 (Ph), 5.2–4.9 (CHCH_3_), 4.3–3.6 (CH_3_O), 3.8–2.2 (CH), 3.7–1.4 (CH_2_), 1.1–0.4 (CH_3_); ^13^C NMR: *δ* 165–163 (C = O), 143–121 (Ph), 118–110 (CN), 71–68 (CH), 60–53 (OCH_3_), 45–40 (CH_2_) 39–32 (CH), 22–18 (CH_3_); FTIR (cm^−1^): 3087–2839 (m, C-H), 2231 (m, CN), 1734 (s, C = O), 1228 (s, C-O-CH_3_), 897 (s, C-H out of plane). Anal. Calcd. for N, 2.32.

#### 2.3.4. Styrene – isopropyl 2-cyano-3-(2-ethoxyphenyl)-2-propenoate copolymer

Yield 14.7%; ^1^H NMR *δ* 7.6–6.0 (Ph), 5.2–4.9 (CHCH_3_), 4.3–3.6 (CH_3_O), 3.8–2.2 (CH), 3.7–1.6 (CH_2_), 1.1–0.4 (CH_3_); ^13^C NMR: *δ* 165–163 (C = O), 143–121 (Ph), 118–110 (CN), 71–68 (CH), 60–52 (OCH_2_), 45–40 (CH_2_); 39–32 (CH), 22–18 (CH_3_)_2_, 20–14 (CH_3_); FTIR (cm^−1^): 3074–2829 (m, C-H), 2240 (m, CN), 1736 (s, C = O), 1266 (s, C-O-CH_3_), 962 (s, C-H out of plane). Anal. N, 2.20.

#### 2.3.5. Styrene – isopropyl 2-cyano-3-(3-ethoxyphenyl)-2-propenoate copolymer

Yield 16.7%; ^1^H NMR *δ* 7.6–6.0 (Ph), 5.2–4.9 (CHCH_3_), 4.3–3.6 (CH_3_O), 3.8–2.2 (CH), 3.7–1.6 (CH_2_), 1.1–0.4 (CH_3_); ^13^C NMR: *δ* 165–163 (C = O), 143–121 (Ph), 118–110 (CN), 71–68 (CH), 60–53 (OCH_2_), 44–39 (CH_2_); 38–30 (CH), 21–17 (CH_3_)_2_, 20–13 (CH_3_); FTIR (cm^−1^): 3078–2834 (m, C-H), 2243 (m, CN), 1754 (s, C = O), 1285 (s, C-O-CH_3_), 887 (s, C-H out of plane). Anal. for N, 2.66.

#### 2.3.6. Styrene – isopropyl 2-cyano-3-(4-ethoxyphenyl)-2-propenoate copolymer

Yield 12.2%; ^1^H NMR *δ* 7.6–6.0 (Ph), 5.3–4.9 (CHCH_3_), 4.3–3.5 (CH_3_O), 3.8–2.1 (CH), 3.7–1.6 (CH_2_), 1.1–0.4 (CH_3_); ^13^C NMR: *δ* 165–163 (C = O), 143–121 (Ph), 118–110 (CN), 72–67 (CH), 60–52 (OCH_2_), 44–40 (CH_2_) 39–31 (CH), 22–18 (CH_3_)_2_, 19–13 (CH_3_); FTIR (cm^−1^): 3035–2805 (m, C-H), 2231 (m, CN), 1734 (s, C = O), 1250 (s, C-O-CH_3_), 846, 756 (s, C-H out of plane). Anal. for N, 2.18.

#### 2.3.7. Styrene – isopropyl 2-cyano-3-(4-propoxyphenyl)-2-propenoate copolymer

Yield 14.2%; ^1^H NMR *δ* 7.7–6.1 (Ph), 5.3–4.9 (CH), 4.2–3.7 (CH_2_O), 1.9–1.5 (CH_3_CH_2_CH_2_O), 1.5–1.2 (CHCH_3_), 1.3–0.8 (CH_3_CH_2_CH_2_O); ^13^C NMR *δ* 166–163 (C = O), 142–122 (Ph), 120–110 (CN), 73–67 (CH_3_CH_2_CH_2_O), 68–65 (OCH), 26–21 (CH_3_CH_2_CH_2_O), 24–19 (OCHCH_3_), 12–8 (OCH_2_CH_2_CH_3_); FTIR (cm^−1^): 3035–2805 (m, C-H), 2232 (m, CN), 1734 (s, C = O), 1254 (s, C-O-CH_3_), 756, 829 (s, C-H out of plane). Anal. for N, 2.40.

#### 2.3.8. Styrene – isopropyl 2-cyano-3-(4-butoxyphenyl)-2-propenoate

Yield 13.8%; ^1^H NMR *δ* 7.8–6.0 (Ph), 5.4–4.9 (CH), 4.3–4.0 (C_3_H_7_CH_2_O), 1.9–1.7 (C_2_H_5_CH_2_CH_2_O), 1.7–1.2 (CH_3_CH_2_CH_2_CH_2_O), 1.6–1.3 (CHCH_3_), 1.2–0.7 (CH_3_CH_2_CH_2_CH_2_O); ^13^C NMR *δ* 168–163 (C = O), 141–118 (Ph), 120–112 (CN), 71–68 (C_3_H_7_CH_2_O), 72–66 (CH), 34–31 (C_2_H_5_CH_2_CH_2_O), 24–20 (CHCH_3_), 22–18 (CH_3_CH_2_CH_2_CH_2_O), 17–13 (CH_3_CH_2_CH_2_CH_2_O); FTIR (cm^−1^): 3034–2808 (m, C-H), 2231 (w, CN), 1736 (s, C = O), 1256 (s, C-O-CH_3_), 874 (s, C-H out of plane). Anal. for N, 1.84.

#### 2.3.9. Styrene – propyl 2-cyano-3-(4-hexyloxyphenyl)-2-propenoate copolymer

Yield 12.1%; ^1^H NMR *δ* 7.9–6.0 (Ph), 5.2–4.6 (CH), 4.5–4.1 (CH_2_O), 1.9–1.7 (CH_2_CH_2_O), 1.8–1.4 (CHCH_3_), 1.6–1.3 (CH_2_CH_2_CH_2_O), 1.5–1.2 (CH_2_CH_2_CH_2_CH_2_O), 1.4–1.2 (CH_2_CH_2_CH_2_CH_2_CH_2_O), 1.2–0.6 (CH_3_CH_2_); ^13^C NMR *δ* 168–163 (C = O), 141–120 (Ph), 118–114 (CN), 72–67 (CH_2_O), 68–65 (CH), 33–28 (CH_2_CH_2_CH_2_CH_2_O), 30–27 (CH_2_CH_2_O), 28–24 (CH_2_CH_2_CH_2_O), 23–20 (CH_3_CH_2_), 22–19 (CHCH_3_), 18–13 (CH_3_CH_2_); FTIR (cm^−1^): 3056–2808 (m, C-H), 2241 (w, CN), 1734 (s, C = O), 1259 (s, C-O-CH_3_), 937 (s, C-H out of plane). Anal. N, 1.86.

## 3. Results and discussion

### 3.1. Synthesis of monomers

**Scheme 1.**
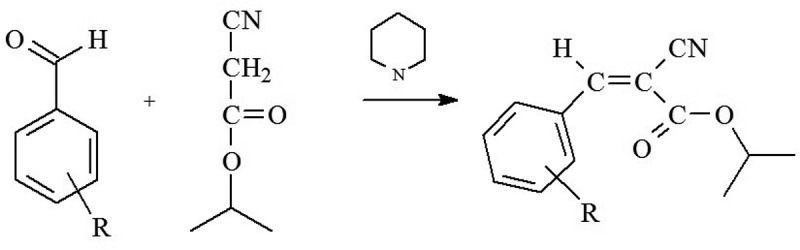
Synthesis of isopropyl 2-cyano-3-(R-phenyl)-2-propenoates where R is 2-methoxy, 3-methoxy, 4-methoxy, 2-ethoxy, 3-ethoxy, 4-ethoxy, 4-propoxy, 4-butoxy, 4-hexyloxy.

All isopropyl 2-cyano-3-phenyl-2-propenoate (ICPP) compounds were synthesized by Knoevenagel condensation [21] of appropriate benzaldehydes with isopropyl cyanoacetate, catalyzed by base, piperidine (Scheme 1).

The condensation reaction yielded crystalline products. No stereochemical analysis of the novel alkoxy ring-substituted ICPP was performed since no stereoisomers (*E* or/and *Z*) of known configuration were available.

### 3.2. Homopolymerization

An attempted homopolymerization of the ICPP compounds in the presence of ABCN did not produce any polymer as indicated by the lack of a precipitate in methanol. The inability of the monomers to polymerize is associated with steric difﬁculties encountered in homopolymerization of 1,1- and 1,2-disubstituted ethylenes [8]. Homopolymerization of ST under conditions identical to those in copolymerization experiments yielded 18.3% of polystyrene, when polymerized for 30 min.

### 3.3. Copolymerization

**Scheme 2.**
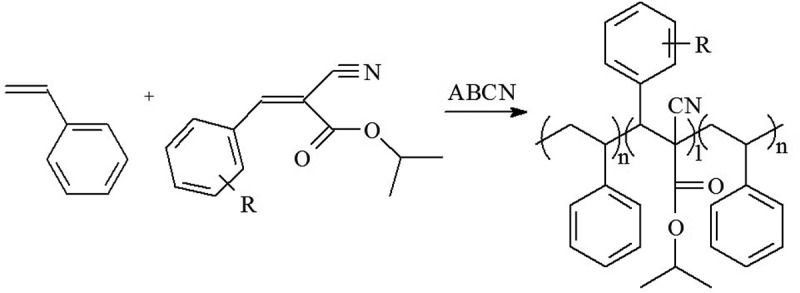
Copolymerization of ST and the alkoxy ring-substituted isopropyl 2-cyano-3-phenyl-2-propenoates, RPhCH = C(CN)CO_2_CH(CH_3_)_2_. R is 2-methoxy, 3-methoxy, 4-methoxy, 2-ethoxy, 3-ethoxy, 4-ethoxy, 4-propoxy, 4-butoxy, 4-hexyloxy.

The novel synthesized ICPP compounds copolymerized readily with ST under free-radical conditions (Scheme 2) forming white flaky precipitates when their solutions were poured into methanol.

The conversion of the copolymers was kept below 20% to minimize compositional drift (Table 1). Nitrogen elemental analysis showed that between 18.0 and 28.0 mol% of ICPP is present in the copolymers prepared at ST/ICPP = 3 (mol), which is indicative of relatively high reactivity of the ICPP monomers towards ST radical which is typical of alkoxy ring-substituted esters of 2-cyano-3-phenyl-2-propenoates [12–18]. Since ICPP monomers do not homopolymerize, the most likely structure of the copolymers would be isolated ICPP monomer units alternating with short ST sequences (Scheme 2).

**Table 1.** Molecular characteristics of ST-ICPP copolymers^a^.

R	Conversion %	Nitrogen wt%	% mole ST	% mole ICPP	M_W_^b^ kD
2-CH_3_O	16.2	2.70	72.5	27.5	72.3
3-CH_3_O	12.3	2.00	81.4	18.6	65.6
4-CH_3_O	13.2	2.32	77.5	22.5	63.4
2-C_2_H_5_O	14.7	2.20	78.4	21.6	61.3
3-C_2_H_5_O	16.7	2.66	72.0	28.0	59.4
4-C_2_H_5_O	12.2	2.18	78.7	21.3	62.7
4-C_3_H_7_O	14.2	2.40	74.9	25.1	62.2
4-C_4_H_9_O	13.8	1.84	82.0	18.0	62.8
4-C_6_H_13_O	12.1	1.86	80.8	19.2	64.9

^a^Conditions: ST/ICPP: 3 (mol)/Toluene/70ºC/5 hrs.

^b^by GPC in THF

The copolymers prepared in the present work are all soluble in ethyl acetate, THF, DMF and CHCl_3_ and insoluble in methanol, ethyl ether, and petroleum ether.

**Figure 1.**
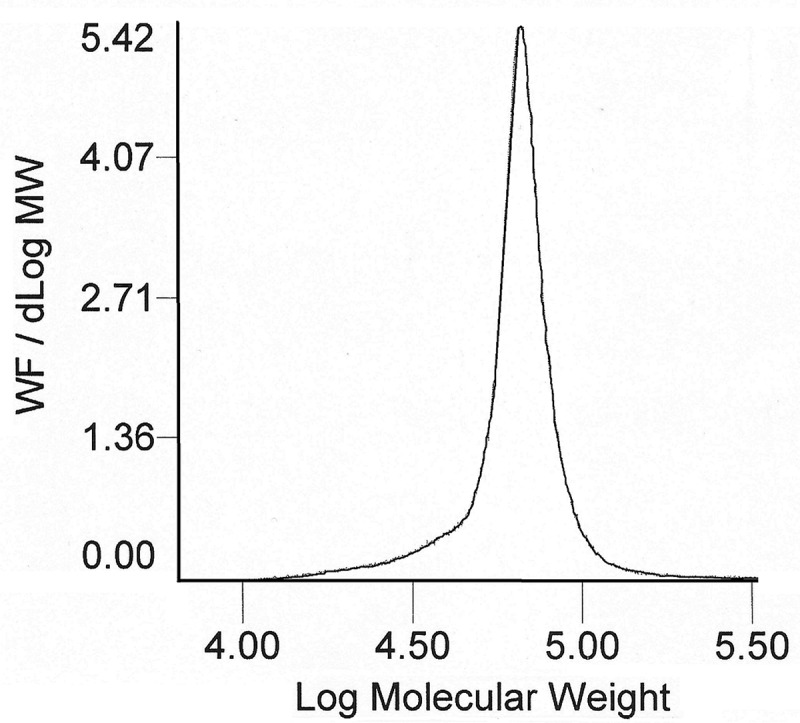
GPC data for P(ST-co-ICPP) copolymer (R = 2-Metoxy).

The molecular weights were measured by GPC in THF. According to GPC analysis the copolymers had weight-average molecular masses 59.4 to 72.3 kD (Table 1). The GPC traces of ST-ring-substituted ICPP copolymers displayed a unimodal peak (Figure 1) characteristic of other ST copolymers with alkoxy ring-substituted propenoate esters [12–18].

Relative reactivities of ST and the ICPP monomers in the copolymerization can be estimated by application of the copolymerization equation for the terminal copolymerization model [8]

(1) $$m_{1}/m_{2}\;=\;[M_{1}]\;(r_{1}\;[M_{1}]\;+\;[M_{2}]\;([M_{1}]\;+\;r_{2}\;[M_{2}]$$

where *m*_1_ and *m*_2_ are mole fractions of ST and ICPP monomer units in the copolymer, [M_1_] and [M_2_] are concentrations of ST and an ICPP in the monomer feed, and *r*_1_ and *r*_2_ are monomer reactivity ratios, *r*_1_ = *k*_ST-ST_/*k*_ST-ICPP_ and *r*_2_ = *k*_ICPP-ICPP_/*k*_ICPP-ST_. In the absence of self-propagation of ICPP monomers (*k*_ICPP-ICPP_ = 0, *r*_2_ = 0), the Equation (1) yields

(2) $$m_{1}/m_{2}\;=\;r_{1}\;([M_{1}]\;/[M_{2}])\;+\;1$$

Equation (2) assumes a minimal copolymer compositional drift during a copolymerization reaction, i.e., a low conversion. The fact that ICPP monomers do not self-propagate allows the use of Equation (2) for a single-point estimation of the relative reactivity of ICPP monomers with respect to ST; it is represented by the 1/*r*_1_ = *k*_ST-ICPP_/*k*_ST-ST_ ratio (the rate constant ratio of attaching an ICPP molecule vs. a ST molecule to a ST-ending growing polymer chain). Thus relative reactivities (1/*r*_1_) for the ICPP monomers decrease in the following row R = 3-ethoxy (1.91) >2-methoxy (1.84) >4-propoxy (1.51) >4-methoxy (1.23) >2-ethoxy (1.14) >4-ethoxy (1.12) >4-hexyloxy (0.94) >3-methoxy (0.89) >4-butoxy (0.84). These ratios indicate that the alkoxy ring-substituted ICPP monomers are slightly more reactive than styrene in the addition to a ST-ended polymer radical. The above reactivities of isopropyl cyanophenylpropenoates are relatively close to those of methyl cyanophenylpropenoates [12–14], (1/*r*_1_) 3-methoxy (2.83) >3-ethoxy (1.50) >2-ethoxy (1.30) >4-ethoxy (0.94) >4-propoxy (0.83) >2-methoxy (0.70) >4-butoxy (0.84) >4-methoxy (0.59) >4-hexyloxy (0.30) and propyl cyanophenylpropenoates [17] where the order of relative reactivity (1/*r*_1_) is 3-ethoxy (2.06) >-methoxy (2.02) >4-methoxy (1.72) >2-methoxy (1.33) >4-ethoxy (1.12) >4-butoxy (1.07) >2-ethoxy (0.95) >4-propoxy (0.93). Reactivities of alkoxy ring-substituted ICPP monomers are higher than those of ethyl cyanophenylpropenoates [15,16], 3-methoxy (0.88) >2-methoxy (0.68) >3-ethoxy (0.66) >4-hexyloxy (0.42) >4-methoxy (0.40) >4-propoxy (0.34) >4-ethoxy (0.25) >4-butoxy (0.20) and lower than those of alkoxy ring-substituted butyl cyanophenylpropenoates [18] 2-cyano-3-phenyl-2-propenoates: 4-methoxy (6.56) >3-methoxy (2.97) >2-methoxy (2.72) >4-butoxy (2.20) >3-ethoxy (2.18) >4-propoxy (2.15) >4-hexyloxy (1.78) >4-ethoxy (1.66) >2-ethoxy (1.48).

### 3.4. Copolymer structure

The structure of ST-ICPP copolymers was characterized by FTIR and NMR spectroscopy. A comparison of the spectra of the monomers, copolymers and polystyrene shows, that the reaction between the ICPP monomers and ST is a copolymerization. FTIR spectra of the copolymers show overlapping bands in 3350–2680 cm^−1^ region corresponding to C-H stretch vibrations. The bands for the ICPP monomer unit are 2240–2228 (w, CN), 1756–1710 (s, C = O), and 1280–1210 cm^−1^ (m, C-O). Phenyl rings of both monomers show ring stretching bands at 1516–1320 cm^−1^ as well as a doublet 778–610 cm^−1^, associated with C-H out-of-plane deformations. These bands can be readily identified in styrene copolymers with TSE monomers containing cyano and carbonyl groups.

**Figure 2.**
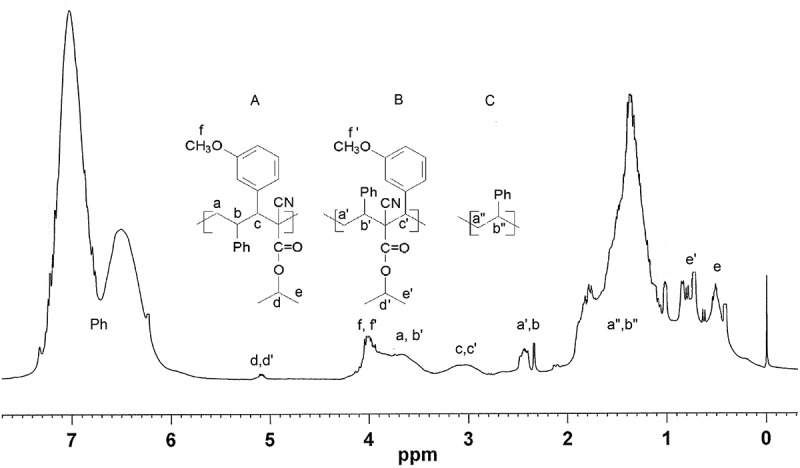
The 300 MHz ^1^H NMR spectrum of P(ST-co-ICPP) copolymer (R = 3-OCH_3_).

Our earlier microstructure analysis of ST copolymers with 2-phenyl-1,1-dicyanoethene [22], as deduced from high resolution ^1^H and ^13^C NMR spectroscopy (DEPT, HETCOR, NOESY and JMODXH), demonstrated the formation of both head-to-tail and head-to-head alternating monomer structures, as well as short ST sequences. The 300 MHz ^1^H NMR spectrum of ST copolymer with isopropyl 2-cyano-3-(3-methoxyphenyl)-2-propenoate is presented in Figure 2.

The ^1^H NMR spectra of the ST-ICPP copolymers show a broad double peak in a 7.5–5.7 ppm region corresponding to phenyl ring protons. A resonance at 5.3–5.0 ppm is assigned to the methineoxy proton of isopropyl ester in both head-to-tail, H-T (structure A) and head-to-head, H-H (structure B) alternating ST-ICPP structures. A signal at 4.2–3.8 ppm is assigned is assigned to the ICPP methoxy protons. Broad overlapping resonances at 3.9–3.3 ppm are assigned to the methylene protons of H-T structures and methine protons of ST in H-H structures. Overlapping resonances at 3.5–2.5 ppm are assigned to mithine protons of ICPP in both A and B structures. Resonance in 2.4–210 is assigned to mithine proton in A structure and methylene protons of ST monomer unit in B structure close to the ICPP, which are more subjected to deshielding than the ones in polystyrene (structure C). A broad resonance peak in 2.0–0.9 ppm range is attributed to the methine and methylene protons of ST monomer sequences, whereas the absorption in 1.0–0.2 ppm is assigned to methyl groups of ICPP isopropyl ester.

**Figure 3.**
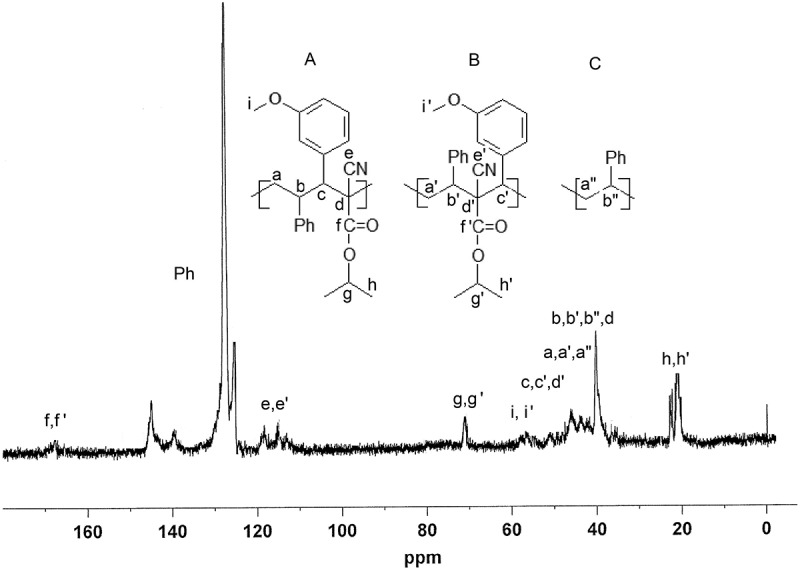
The 75 MHz ^13^C NMR spectrum of P(ST-co-ICPP) copolymer (R = 3-methoxy).

The 75 MHz ^13^C NMR spectrum of ST copolymer with isopropyl 2-cyano-3-(3-methoxyphenyl)-2-propenoate shown in Figure 3 also supports the suggested skeletal structure of the copolymers.

The peak in 172–163 ppm range is assigned to the carbonyl of the ICPP isopropyl group. Quaternary carbons of both phenyls, ST and ICPP, absorb in 144–135 ppm range with the rest of ring carbon resonances at 135–120 ppm. Double nitrile carbon signal in both H-T (A) and H-H (B) alternating ST-ICPP structures is observed in 120–110 ppm range. Signal at 72–68 ppm is assigned methineoxy carbon of isopropyl ester in both A and B structures. Absorption at 59–55 ppm is assigned to ICPP methoxy carbons. Overlapping resonances at 53–35 ppm are assigned to methine and quaternary carbons of ICPP in A and B alternating ST-ICPP structures. Resonances in 50–42 ppm are assigned to ST methylene carbons in all three structures, A,B, and C. Signals 23–15 ppm are assigned to methyl carbons of isopropyl group of ICPP. Broadening of the NMR signals in the spectra of the copolymers (independent of magnetic field strength) is apparently associated with head-to-tail and head-to-head structures, which formed though the attack of a styrene-ended radical on both sides of ICPP monomer or/and participation of both free monomers and monomer donor-acceptor complexes [8] The FTIR and NMR data showed that these are true copolymers, composed of both ICP and ST monomer units.

### 3.4. Thermal behavior

**Figure 4.**
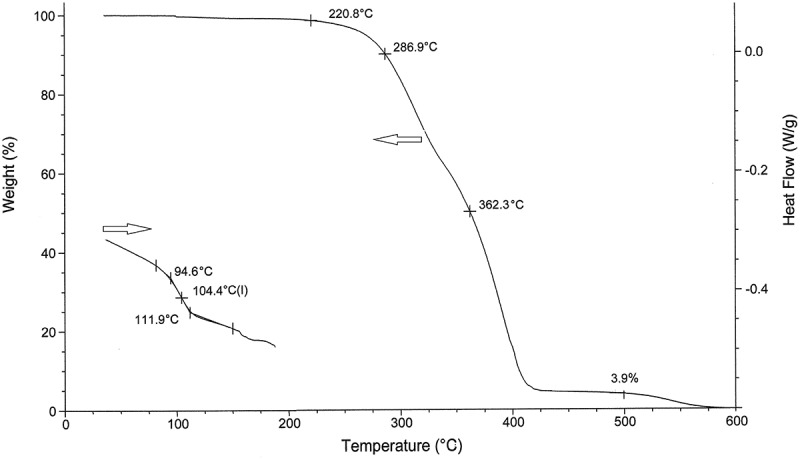
DSC and TGA of P(ST-co-ICPP) copolymer (R = 3-methoxy).

Thermal transitions of the ST-ICPP copolymers were analyzed by differential scanning calorimetry. All the copolymers were amorphous and show no crystalline DSC endotherm on repeated heating and cooling cycles. The glass transition temperatures *T*_g_ of the copolymers were measured by DSC. The second heating results were obtained in all cases so that the samples become drier without ‘thermal memory’. Figure 4 shows DSC trace of ST copolymer with isopropyl 2-cyano-3-(3-methoxyphenyl)-2-propenoate. Table 2 shows glass transition values for the ST-ICPP copolymers prepared in this work with no correlation to the size and position of the ICPP alkoxy ring substitution apparently due to non-uniform composition, monomer unit distribution, and/or molecular weight and MWD. A single *T*_g_ value was observed for all the copolymers with values close or higher than polystyrene (104ºC) [23]. We compared glass transitions of styrene copolymers with different esters of cyanophenylpropenoates. Thus *T*_g_ values of ST-ICPP copolymers are lower than those of the ST copolymers with methyl cyanophenylpropenoates [12–14] (ºC), 3-methoxy (220) >4-methoxy (210) >4-ethoxy (179) >2-methoxy (172) >4-butoxy (147), 4-propoxy (131) >3-ethoxy (126) >2-ethoxy (120) >4-hexyloxy (110), ethyl cyanophenylpropenoates [15,16], 3-methoxy (174) >2-methoxy (161) >4-methoxy (146) >4-butoxy (136) >4-propoxy (127) >4-ethoxy (125) >3-ethoxy (115) >4-hexyloxy (96), and propyl cyanophenylpropenoates [17] 2-methoxy (153) >2-ethyl (142) >3-methoxy (136) >4-ethoxy (125) >3-ethyl (122) >4-butyl (118) >4-methoxy (110) >4-propyl (107), and higher than butyl cyanophenylpropenoates [18], 3-ethoxy (80) >3-methoxy (78) >4-propyl (73) >2-methoxy (71) >2-ethoxy (70) >4-ethoxy (67) >4-hexyloxy (64) >4-methoxy (62) >4-butoxy (60).

**Table 2.** Thermal behavior of P(ST-co-ICPP) copolymers.

TGA					
R	T_g_ ºC	Onset of decomp., ºC	10% wt loss, ºC	50% wt loss, ºC	Residue at 500 ºC, wt%
2-CH_3_O	102	231	276	325	3.4
3-CH_3_O	104	221	287	362	3.9
4-CH_3_O	105	246	294	334	3.1
2-C_2_H_5_O	103	242	290	334	2.9
3-C_2_H_5_O	104	216	287	363	3.8
4-C_2_H_5_O	101	210	285	337	2.6
4-C_3_H_7_O	98	232	277	336	3.2
4-C_4_H_9_O	96	174	234	283	3.3
4-C_6_H_13_O	93	205	282	351	3.1

Information on thermal stability of the copolymers was obtained from thermogravimetric analysis (Table 2). Figure 4 shows TGA of the P(ST-co-ICPP) copolymer. The copolymer has lower thermal stability than is reported for polystyrene (PS) [24], the onset of decomposition at 221ºC (PS 350ºC), 10% weight loss at 287ºC (PS 425ºC), 50% weight loss at 362ºC (PS 428ºC). Lower thermal stability of the ST-ICPP copolymers apparently associated with presence of ICPP quaternary carbon in the chain similarly to poly-alpha-methylstyrene [25]. Decomposition of the copolymers in nitrogen occurred in two steps, first in the 250–500ºC range with residue (2.6–3.9% wt), which then decomposed in the 500–800ºC range. The decomposition products were not analyzed in this study, and the mechanism has yet to be investigated.

## 4. Conclusions

Novel trisubstituted ethylenes, alkoxy ring-substituted isopropyl 2-cyano-3-phenyl-2-propenoates were prepared and copolymerized with styrene. The compositions of the copolymers were calculated from nitrogen analysis and the structures were analyzed by IR, H^1^ and ^13^C NMR. The thermal gravimetric analysis indicated that the copolymers decompose in two steps, first in the 250–500°C range with residue (2.6–3.9%wt), which then decomposed in the 500–800ºC range.
